# Supplementary material for: COZOID: contact zone identifier for visual analysis of protein-protein interactions
Source: BMC Bioinformatics. 2018 Apr 6;19:125. doi: 10.1186/s12859-018-2113-6 (PMC5889581; doi:10.1186/s12859-018-2113-6)
Supplement: Supplementary file 2 — Software build. Executable binary file of the software tool. (ZIP 118,784 kb) [file 12859_2018_2113_MOESM2_ESM.zip › cozoid/platform/docs/testng-6.5.1-javadoc.zip!/org/testng/ISuiteListener.html]

ISuiteListener (TestNG)


---


|  |  |  |
| --- | --- | --- |
| **Overview** \| **Package** \| **Class** \| **Tree** \| **Deprecated** \| **Index** \| **Help** | |  |
| **PREV CLASS**   **NEXT CLASS** | **FRAMES**    **NO FRAMES**     **All Classes** |  |
| SUMMARY: NESTED \| FIELD \| CONSTR \| METHOD | DETAIL: FIELD \| CONSTR \| METHOD |  |


---


## org.testng Interface ISuiteListener

**All Superinterfaces:**: ITestNGListener

---

``` public interface ISuiteListener extends ITestNGListener ```

Listener for test suites.

**Author:**
:   Cedric Beust, Aug 6, 2004

---

| **Method Summary** | |
| --- | --- |
| `void` | `onFinish(ISuite suite)`             This method is invoked after the SuiteRunner has run all the test suites. |
| `void` | `onStart(ISuite suite)`             This method is invoked before the SuiteRunner starts. |

**Method Detail**

### onStart

```
void onStart(ISuite suite)
```

:   This method is invoked before the SuiteRunner starts.

---


### onFinish

```
void onFinish(ISuite suite)
```

:   This method is invoked after the SuiteRunner has run all
    the test suites.


---


|  |  |  |
| --- | --- | --- |
| **Overview** \| **Package** \| **Class** \| **Tree** \| **Deprecated** \| **Index** \| **Help** | |  |
| **PREV CLASS**   **NEXT CLASS** | **FRAMES**    **NO FRAMES**     **All Classes** |  |
| SUMMARY: NESTED \| FIELD \| CONSTR \| METHOD | DETAIL: FIELD \| CONSTR \| METHOD |  |


---
